# The Work Performed by the Diagnosis Laboratory of the Department of Health in Connection with Ehrlich’s Diazo Reaction during 1902

**Published:** 1903-09

**Authors:** J. S. Billings


### The Work Performed by the Diagnosis Laboratory of the Department of Health in Connection with Ehrlich's Diazo Reaction During 1902.

J. S. BILLINGS, JR.

The writer's conclusions are: (1) The examination of the urine in cases of suspected typhoid fever is of value, provided that its limitations are recognized. (2) While not so absolutely pathognomonic of typhoid fever, yet the diazo reaction is even more constantly present in that disease than the Widal reaction, so that its absence at a period when it should be present, if the case is one of typhoid fever, is of considerable value in making a negative diagnosis. (3) In a majority of instances the diazo reaction is present in the urine at least forty-eight hours earlier than the Widal reaction in the blood. (4) It disappears much earlier than the Widal reaction, however, and engative results obtained later than the second week are of little or no value. (5) "Doubtful reactions" have slight significance.—*New York Medical Journal*, April 18, 1903.

